# Supplementary material for: A nuclear targeting approach enables efficient transgenesis in the milkweed bug Oncopeltus fasciatus
Source: bioRxiv. 2026 Jul 31:2026.07.30.741915. Preprint. [Version 2] doi: 10.64898/2026.07.30.741915 (PMC13484257; doi:10.64898/2026.07.30.741915)
Supplement: Supplementary file 1 [file NIHPP2026.07.30.741915v2-supplement-1.pdf]

bioRxiv preprint doi: https://doi.org/10.64898/2026.07.30.741915; this version posted July 31, 2026. The copyright holder for this preprint (which was not certified by peer review) is the author/funder, who has granted bioRxiv a license to display the preprint in perpetuity. It is made available under a CC-BY 4.0 International license.

Supplementary Figures

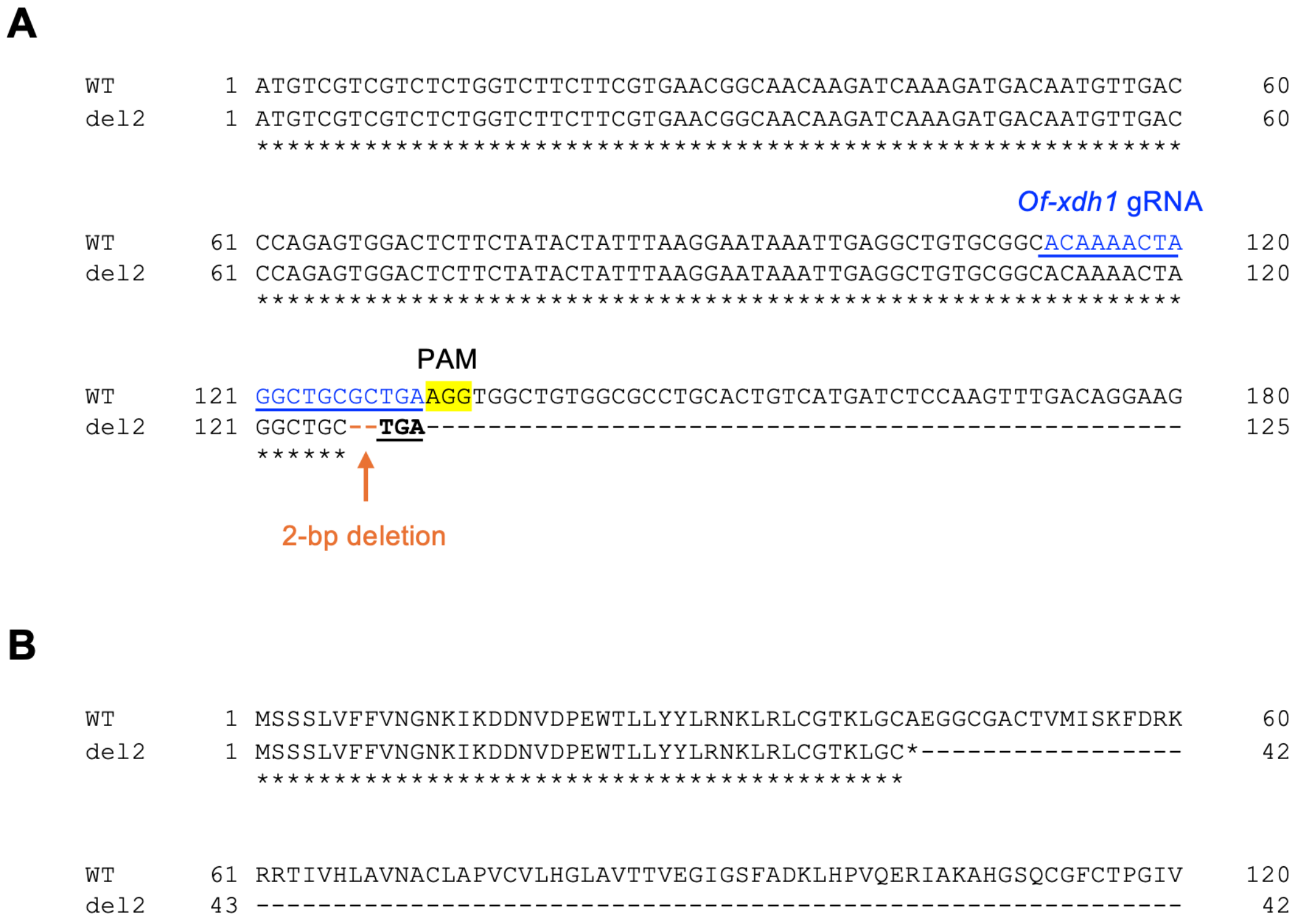

Figure S1. Nucleotide and amino acid sequence of *Of-xdh1* in the *xdh1*$^{\Delta 2}$ mutant. (A) Alignment of the nucleotide sequences of *Of-xdh1* around the gRNA target site of WT and *xdh*$^{\Delta 2}$ strains. Note the presence of a 2-bp deletion in the *xdh1*$^{\Delta 2}$ allele (shown in orange). The premature stop codon induced by this deletion is indicated in bold black. (B) Alignment of the amino acid sequences of *Of-xdh1* of the WT and *xdh1*$^{\Delta 2}$ strains. The 2-bp deletion caused a frameshift mutation in the *xdh1*$^{\Delta 2}$ allele.

bioRxiv preprint doi: https://doi.org/10.64898/2026.07.30.741915; this version posted July 31, 2026. The copyright holder for this preprint (which was not certified by peer review) is the author/funder, who has granted bioRxiv a license to display the preprint in perpetuity. It is made available under a CC-BY 4.0 International license.

A

```
WT      1 ATGGAGCCCGACAGCAATTGCAGATCTTCCGAGATCCAGAA-ATGTGGAGAGCAAAGTGG    59
v1      1 ATGGAGCCCGACAGCAATTGCAGATCTTCCGAGATCCAGAAGATGTGGAGAGCAAAGTGG    60
          ***************************************** ******************
                                                   ↑
                                            1-bp insertion

WT     60 AATGCTGTATGGGGATTACCTCCAACTTGACAAAATACTGAAGGCCCAAAGATTGCTGAG   119
v1     61 AATGCTGTATGGGGATTACCTCCAACTTGA------------------------------    90
          ******************************
```

B

```
WT      1 MEPDSNCRSSEIQKCGEQSGMLYGDYLQLDKILKAQRLLSESNNATVMDEHLFIVTHQGN    60
v1      1 MEPDSNCRSSEIQKMWRAKWNAVWGLPPT*------------------------------    29
          **************

WT     61 KLNSTQEMTNEGLNERQTLDVVKRLNRIVLILKLLVEQVMILETMTPLDFMEFRDYLCPA   120
v1     29 ------------------------------------------------------------    29

WT    121 SGFQSAQFRLIENKLGVKTEHRVRSFQHYSTVFERDKEALNEIKKSETEQSLADLIEQWL   180
v1     29 ------------------------------------------------------------    29

WT    181 ERTPGLDENGFDFWGKYIKNVEFLLQHYYEKAMKSNSETEKTTKLAEYEKKKTIFNSVLN   240
v1     29 ------------------------------------------------------------    29

WT    241 KDMHDLFVQRGERRFTHKAFQGAIMITFYRDEPRFSQPHQILTLLMDIDSLVTKWRLCSK   300
v1     29 ------------------------------------------------------------    29

WT    301 TWINYYNDKYKVLLISDRYKCFLDLFNLSSFLLPRNFIPPLSSSVRNQINIHTWNTKKQT   360
v1     29 ------------------------------------------------------------    29

WT    361 EDHEED*                                                         366
v1     29 ------                                                          29
```

Figure S2. Nucleotide and amino acid sequence of *Of-v* in the v^1 mutant. (A) Alignment of the nucleotide sequences of *Of-v* around the gRNA target site of wild type (WT) and v^1 strains. Note the presence of a 1-bp insertion in the v^1 allele (shown in red). The premature stop codon induced by this insertion is indicated in bold black. Further details are described in (Reding and Pick, 2023). (B) Alignment of the amino acid sequences of *Of-v* of the WT and v^1 strains. The 1-bp insertion caused a frameshift mutation in the v^1 allele (shown in red).

bioRxiv preprint doi: https://doi.org/10.64898/2026.07.30.741915; this version posted July 31, 2026. The copyright holder for this preprint (which was not certified by peer review) is the author/funder, who has granted bioRxiv a license to display the preprint in perpetuity. It is made available under a CC-BY 4.0 International license.

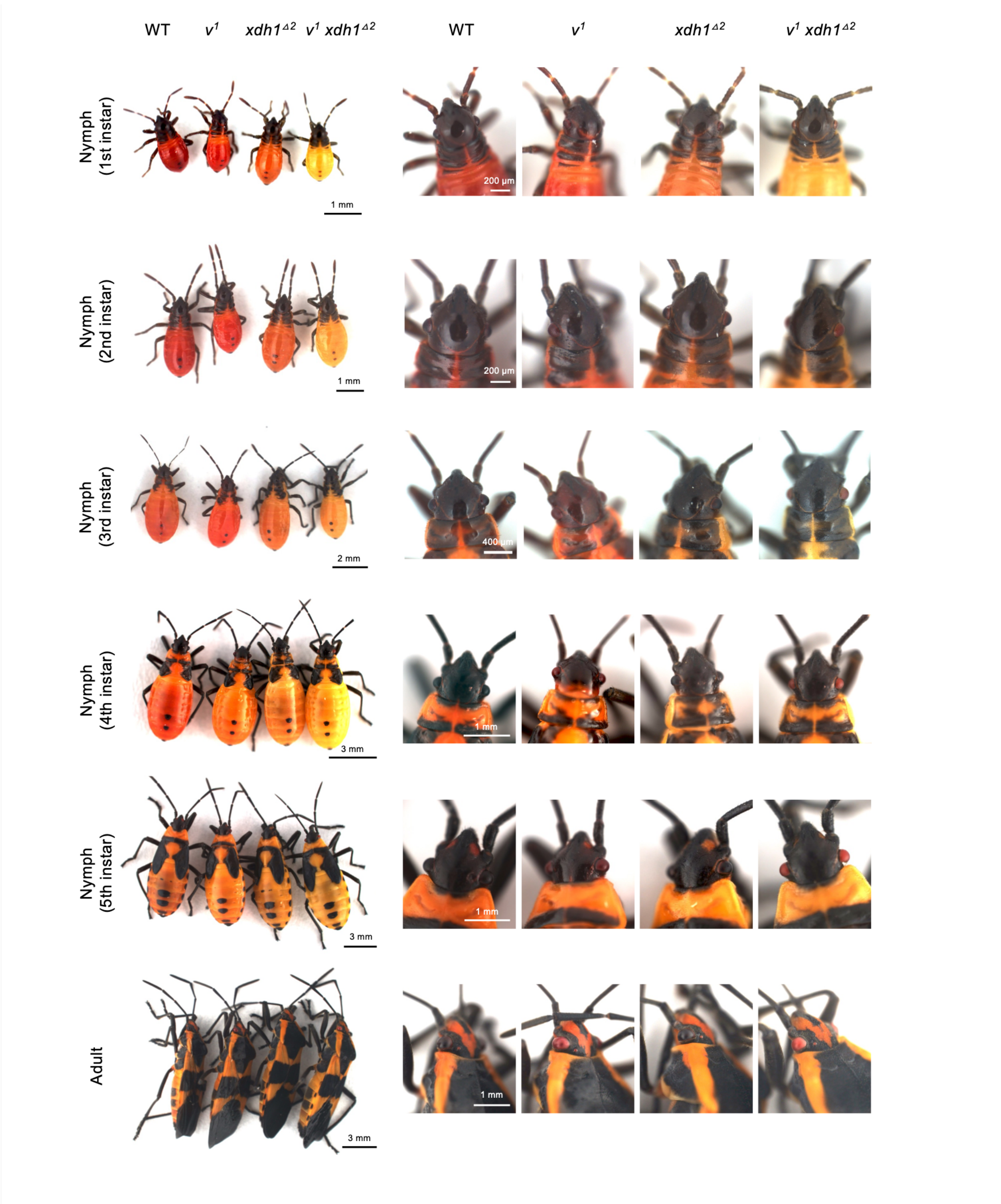

Figure S3. Comparisons of phenotypes of wild type (WT), *v*^{1}, *xdh1*$^{\Delta 2}$, and *v*^{1} *xdh1*$^{\Delta 2}$ throughout postembryonic development. All images of nymphs and close-up views (bottom right) are shown in dorsal view; low-magnification adult image (bottom left) is shown in lateral view. Anterior is up in all images.

bioRxiv preprint doi: https://doi.org/10.64898/2026.07.30.741915; this version posted July 31, 2026. The copyright holder for this preprint (which was not certified by peer review) is the author/funder, who has granted bioRxiv a license to display the preprint in perpetuity. It is made available under a CC-BY 4.0 International license.

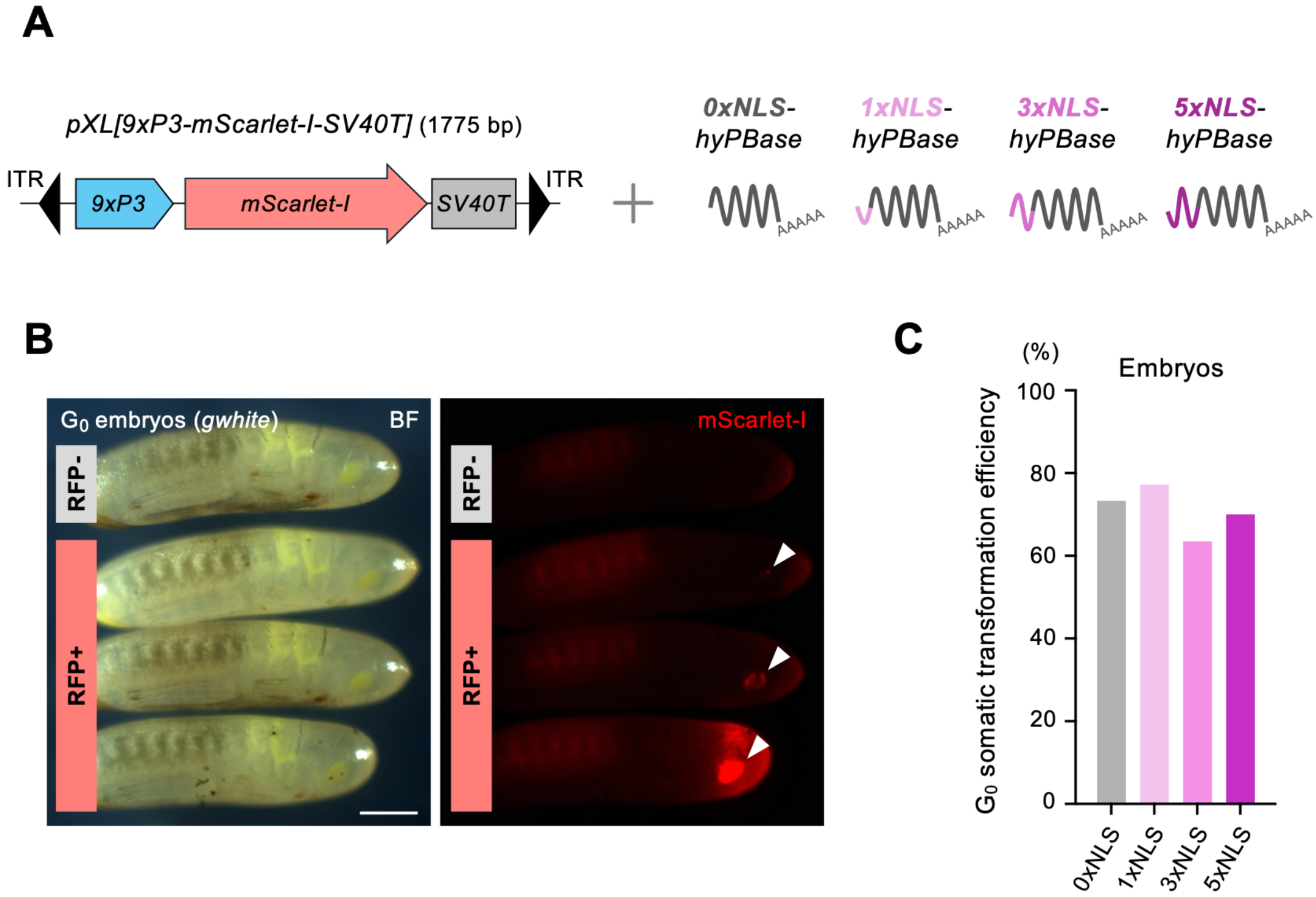

Figure S4. Investigation of a nuclear targeting approach for *piggyBac*-mediated transgenesis in *G. bimaculatus*. (A) Schematic of the *piggyBac* donor vector *pXL[9×P3-mScarlet-I-SV40T]* and *hyPBase* mRNA carrying 0×, 1×, 3×, or 5× NLS. (B) Representative images of G_0 RFP-positive embryos (embryonic stage >13) (Donoughe and Extavour, 2016). Scale bar, 500 µm. Arrowheads indicate eyes. Anterior is to the right. (C) G_0 somatic transformation rate (STR) across 0×, 1×, 3×, or 5×NLS *hyPBase* conditions.

bioRxiv preprint doi: https://doi.org/10.64898/2026.07.30.741915; this version posted July 31, 2026. The copyright holder for this preprint (which was not certified by peer review) is the author/funder, who has granted bioRxiv a license to display the preprint in perpetuity. It is made available under a CC-BY 4.0 International license.

Integration site in 9xP3-EGFP, attP strain 5A

TATTTCAAGAATGCATGCGTCAATTTTACGCAGACTATCTTTCTAGGG**TTAA**TTAAAATTGCGTGGTACGATTTG
TAATCTACAGTTTACCCGGACACTAAATTACAATTTTACGTTTTATTGGCCGAAAATAGTTGTTTTTCTAGACAA
AA

Scaffold407: 476862-476763 (pident = 100%)

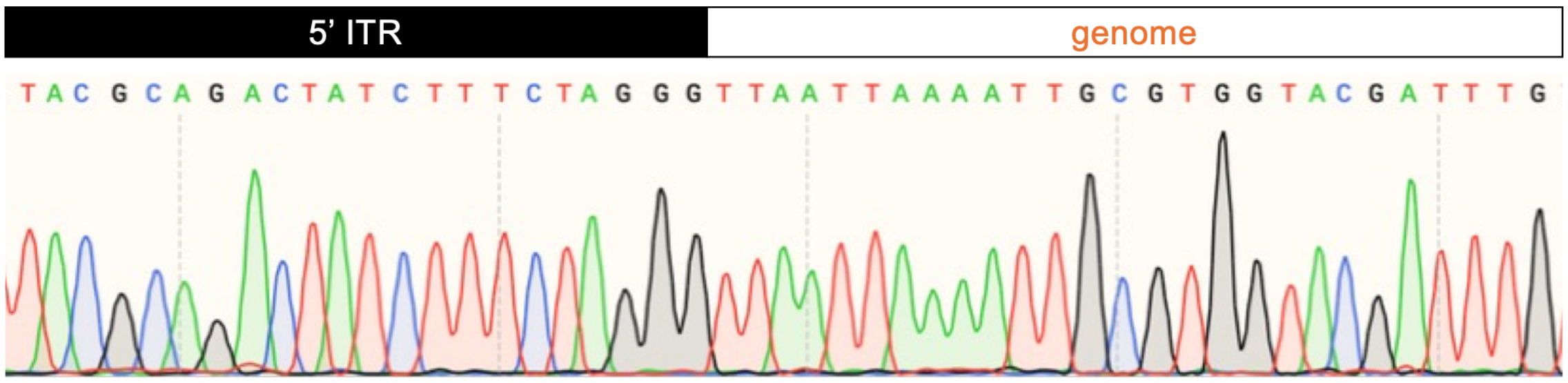

Figure S5. Nucleotide sequences around the transgene integration site in the *9×P3-EGFP*, *attP* strain in *O. fasciatus* as determine by splinkerette PCR.

bioRxiv preprint doi: https://doi.org/10.64898/2026.07.30.741915; this version posted July 31, 2026. The copyright holder for this preprint (which was not certified by peer review) is the author/funder, who has granted bioRxiv a license to display the preprint in perpetuity. It is made available under a CC-BY 4.0 International license.

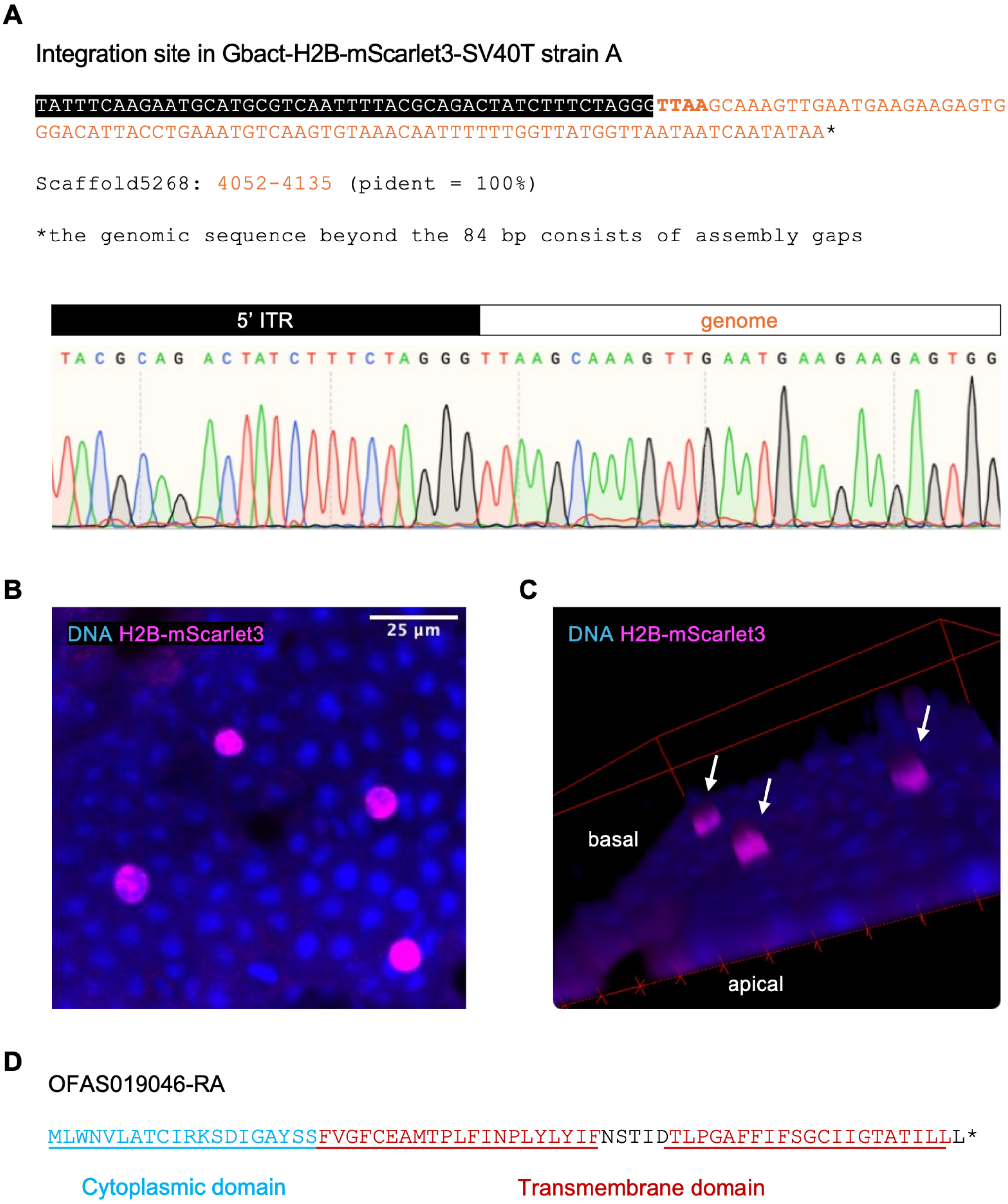

Fig S6. Additional information on the *H2B-mScarlet3-SV40T* strain in *O. fasciatus.* (A) Injection and crossing experiments used to generate the *H2B-mScarlet3-SV40T* strain. (A) Nucleotide sequences around the transgene integration site in the *H2B-mScarlet3-SV40T* strain as determined by splinkerette PCR. (B) Confocal image of the abdominal integument of the *H2B-mScarlet3-SV40T* strain co-stained for DNA. Epidermal nuclei were not labelled with H2B-mScarlet3. (D) 3D reconstruction of the abdominal integument of the H2B-mScarlet3 strain co-stained for DNA, showing that the histone-labelled nuclei with H2B-mScarlet3 were located underneath the epidermal nuclei. (E) Amino acid sequences of the uncharacterized protein (OFAS019046-RA) encoded by the locus containing the transgene insertion site.

bioRxiv preprint doi: https://doi.org/10.64898/2026.07.30.741915; this version posted July 31, 2026. The copyright holder for this preprint (which was not certified by peer review) is the author/funder, who has granted bioRxiv a license to display the preprint in perpetuity. It is made available under a CC-BY 4.0 International license.

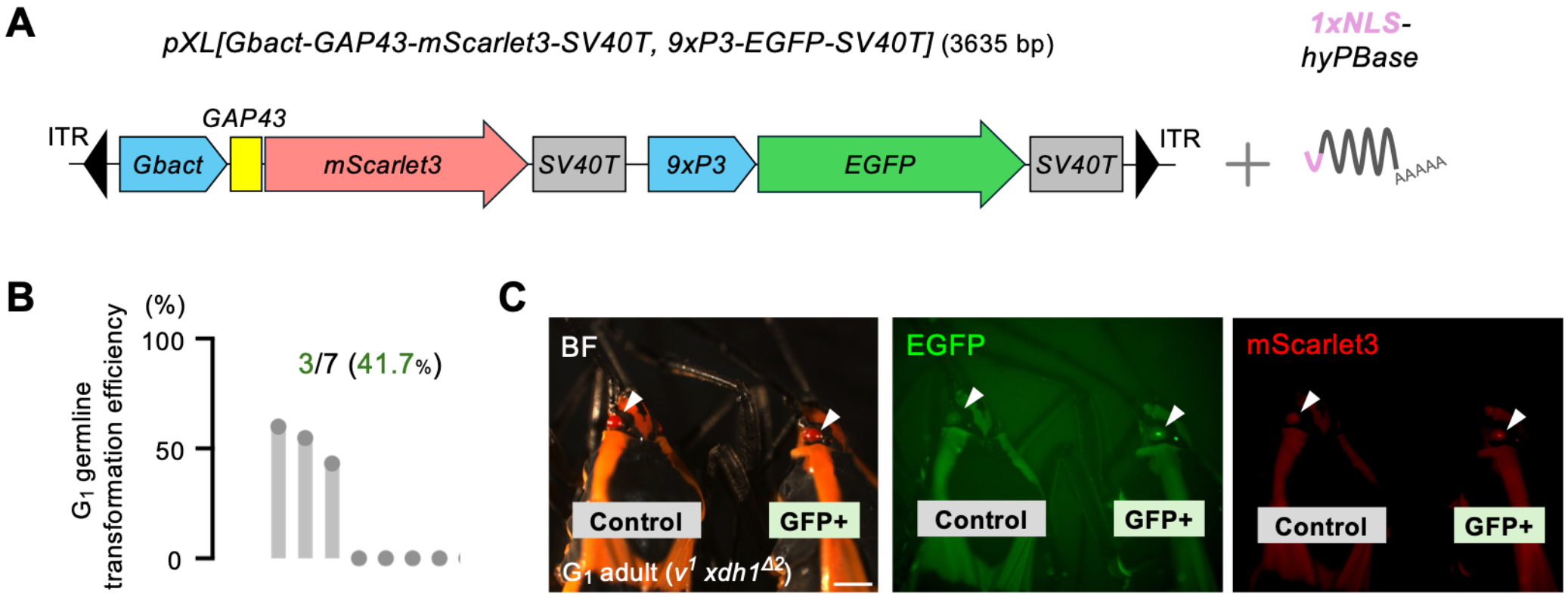

Figure S7. Attempt to generate a membrane-labelled strain using 1×NLS *hyPBase* in *O. fasciatus*. (A) Schematic of the *piggyBac* donor vector *pXL[Gbact-GAP43-mScarlet3-SV40T, 9xP3-EGFP-SV40T]* and *hyPBase* mRNA carrying 1×NLS. (B) G_0 somatic transformation rate (STR). (C) G_1 germ line transformation efficiency (GTE). Each bar represents the proportion of EGFP-positive animals among G_1 progeny from an individual cross. Each dot represents an individual backcross that yielded G_1 progeny. Green numbers indicate the number of crosses yielding heritable transgenes and the corresponding percentages. (D) Representative images of G_1 EGFP-positive adults. Weak mScarlet3 expression in their eyes was also observed. (E) Results of G_1 crossing experiments. Each row indicates the result of a single-pair mating.

bioRxiv preprint doi: https://doi.org/10.64898/2026.07.30.741915; this version posted July 31, 2026. The copyright holder for this preprint (which was not certified by peer review) is the author/funder, who has granted bioRxiv a license to display the preprint in perpetuity. It is made available under a CC-BY 4.0 International license.

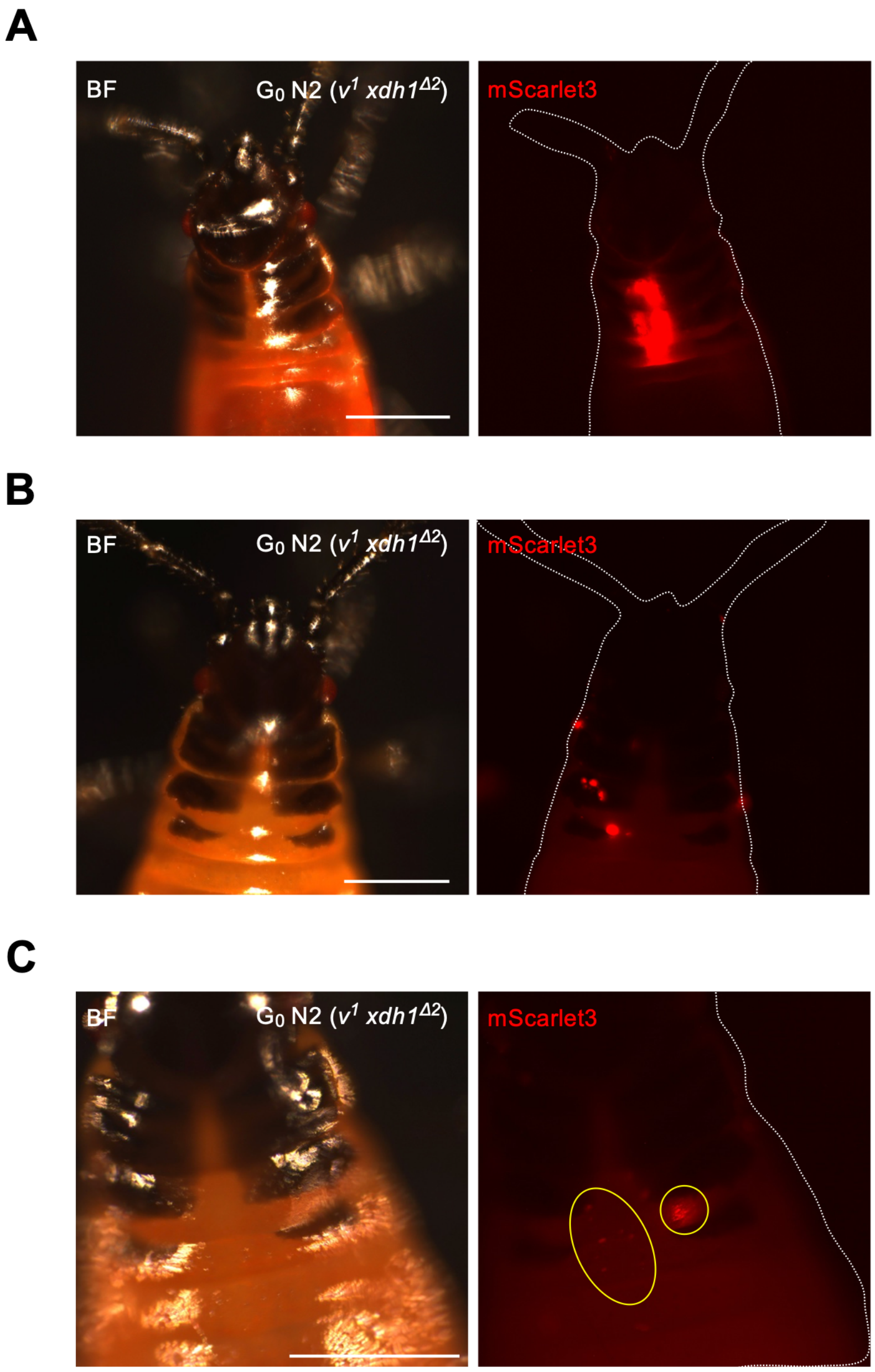

Figure S8. Additional data on Q system application using 1×NLS *hyPBase* in *O. fasciatus*. (A–C) Representative images of G_0 mScarlet3-positive nymphs. White dotted lines indicate animal boundaries. Yellow dotted circles indicate mScarlet3 expression. Scale bars, 500 μm. Anterior is up in (B–D).

Supplementary Tables

Table S1. Detailed results of piggyBac-mediated transgenesis experiments with hyPBase mRNA carrying 0×, 1×, 3×, or 5×NLS in *G. bimaculatus*. Each row shows the results of an independent experiment. Percentages shown in parentheses indicate somatic transformation rates (STR).

Donor plasmid	Transposase (mRNA)	Injected eggs	% of injected eggs survived to screening	Screened G0 embryos	mScarlet-I **+** nymphs	mScarlet-I **-** nymphs
pXL[9xP3-mScarlet-I-SV40T]	0xNLS-hyPBase	194	69.6%	135	99 (73.3%)	36
pXL[9xP3-mScarlet-I-SV40T]	1xNLS-hyPBase	194	70.1%	136	105 (77.2%)	31
pXL[9xP3-mScarlet-I-SV40T]	3xNLS-hyPBase	190	72.1%	137	87 (63.5%)	50
pXL[9xP3-mScarlet-I-SV40T]	5xNLS-hyPBase	192	67.7%	130	91 (70.0%)	39

Table S2. Results of crossing experiments with 0×NLS-hyPBase. F, female. M, male. GTR, germ line transformation rate.

mRNA	donor plasmid
0xNLS-hyPBase#1	9xP3-mScarlet-I-SV40T

G0 indiv#	mScarlet-I expression	sex	crossed with	Screened G1 animals	RFP**+**	RFP**-**	GTR (%)
1	+	M	v^1 $xdh1^{\Delta}$	60	0	60	0
2	+	F	v^1 $xdh1^{\Delta}$	57	0	57	0
3	+	M	v^1 $xdh1^{\Delta}$	57	0	57	0
4	+	F	v^1 $xdh1^{\Delta}$	54	31	23	57.4
5	+	F	v^1 $xdh1^{\Delta}$	57	0	57	0
6	+	F	v^1 $xdh1^{\Delta}$	13	0	13	0

Table S3. Results of crossing experiments with 1×NLS-hyPBase. F, female. M, male. GTR, germ line transformation rate.

mRNA	donor plasmid						
1xNLS-hyPBase#1	9xP3-mScarlet-I-SV40T						
G0 indiv#	mScarlet-I expression	sex	crossed with	Screened G1 animals	RFP**+**	RFP**-**	GTR (%)
1	+	F	v^{1} $xdh1^{\Delta2}$	55	0	55	0
2	+	F	v^{1} $xdh1^{\Delta2}$	45	0	45	0
3	+	F	v^{1} $xdh1^{\Delta2}$	64	0	64	0
4	+	F	v^{1} $xdh1^{\Delta2}$	52	0	52	0
5	+	F	v^{1} $xdh1^{\Delta2}$	53	44	9	83.0
6	+	M	v^{1} $xdh1^{\Delta2}$	sterile	0	0	NA
7	+	M	v^{1} $xdh1^{\Delta2}$	24	11	13	45.8
8	+	M	v^{1} $xdh1^{\Delta2}$	50	0	50	0
9	+	F	v^{1} $xdh1^{\Delta2}$	34	0	34	0
10	+	F	v^{1} $xdh1^{\Delta2}$	43	0	43	0
11	+	F	v^{1} $xdh1^{\Delta2}$	61	54	7	88.5
12	+	M	v^{1} $xdh1^{\Delta2}$	39	18	21	46.2
13	+	M	v^{1} $xdh1^{\Delta2}$	51	0	51	0
14	+	M	v^{1} $xdh1^{\Delta2}$	52	35	17	67.3

15	+	M	v^1 $xdh1^{\Delta2}$	48	0	48	0
16	+	M	v^1 $xdh1^{\Delta2}$	26	0	26	0
17	+	M	v^1 $xdh1^{\Delta2}$	62	0	62	0
18	+	M	v^1 $xdh1^{\Delta2}$	46	6	40	13.0
19	+	M	v^1 $xdh1^{\Delta2}$	50	0	50	0
20	+	M	v^1 $xdh1^{\Delta2}$	sterile	0	0	NA

mRNA	donor plasmid
1xNLS-hyPBase#2	9xP3-mScarlet-I-SV40T

G0 indiv#	mScarlet-I expression	sex	crossed with	Screened G1 animals	RFP**+**	RFP**-**	GTR (%)
1	+	M	v^1 $xdh1^{\Delta2}$	51	0	51	0
2	+	F	v^1 $xdh1^{\Delta2}$	55	0	55	0
3	+	F	v^1 $xdh1^{\Delta2}$	60	60	0	100.0
4	+	F	v^1 $xdh1^{\Delta2}$	56	0	56	0
5	+	F	v^1 $xdh1^{\Delta2}$	52	0	52	0
6	+	M	v^1 $xdh1^{\Delta2}$	50	0	50	0
7	+	F	v^1 $xdh1^{\Delta2}$	sterile	0	0	NA
8	+	F	v^1 $xdh1^{\Delta2}$	50	50	0	100.0

9	+	M	v^1 $xdh1^{\Delta 2}$	53	53	0	100.0
10	+	M	v^1 $xdh1^{\Delta 2}$	51	0	51	0

Table S4. Results of crossing experiments with 3×NLS-hyPBase. F, female. M, male. GTR, germ line transformation rate.

mRNA	donor plasmid						
3xNLS-hyPBase#1	9xP3-mScarlet-I-SV40T						
G0 indiv#	mScarlet-I expression	sex	crossed with	Screened G1 animals	RFP**+**	RFP**-**	GTR (%)
1	+	M	*v^1 $xdh1^{\Delta2}$*	60	0	60	0
2	+	M	*v^1 $xdh1^{\Delta2}$*	55	0	55	0
3	+	M	*v^1 $xdh1^{\Delta2}$*	3	3	0	100.0
4	+	F	*v^1 $xdh1^{\Delta2}$*	51	33	18	64.7
18	+	M	*v^1 $xdh1^{\Delta2}$*	61	0	61	0
19	+	M	*v^1 $xdh1^{\Delta2}$*	1	0	1	0
20	+	M	*v^1 $xdh1^{\Delta2}$*	57	0	57	0
41	+	M	*v^1 $xdh1^{\Delta2}$*	30	0	30	0

mRNA	donor plasmid						
3xNLS-hyPBase#2	9xP3-mScarlet-I-SV40T						
G0 indiv#	mScarlet-I expression	sex	crossed with	Screened G1 animals	RFP**+**	RFP**-**	GTR (%)
18	+	M	*v^1 xdh1$^{\Delta 2}$*	51	0	51	0
19	+	M	*v^1 xdh1$^{\Delta 2}$*	sterile	0	0	NA
20	+	M	*v^1 xdh1$^{\Delta 2}$*	57	0	57	0
21	+	M	*v^1 xdh1$^{\Delta 2}$*	68	0	68	0
22	+	M	*v^1 xdh1$^{\Delta 2}$*	64	64	0	100.0
23	+	M	*v^1 xdh1$^{\Delta 2}$*	36	0	36	0
24	+	F	*v^1 xdh1$^{\Delta 2}$*	34	0	34	0
25	+	F	*v^1 xdh1$^{\Delta 2}$*	23	0	23	0
26	+	F	*v^1 xdh1$^{\Delta 2}$*	died	0	0	NA
27	+	M	*v^1 xdh1$^{\Delta 2}$*	45	0	45	0
28	+	M	*v^1 xdh1$^{\Delta 2}$*	sterile	0	0	NA
29	+	F	*v^1 xdh1$^{\Delta 2}$*	50	50	0	100.0
30	+	F	*v^1 xdh1$^{\Delta 2}$*	54	23	31	42.6
31	+	F	*v^1 xdh1$^{\Delta 2}$*	51	51	0	100.0
40	+	F	*v^1 xdh1$^{\Delta 2}$*	11	11	0	100.0

Table S5. Results of crossing experiments for attP strains. The integration site was determined for the line derived from G1 individual 8A. F, female. M, male. GTR, germ line transformation rate.

mRNA	donor plasmid						
3xNLS-hyPBase#1	9xP3-EGFP-SV40T;attP						
G0 indiv#	EGFP expression	sex	crossed with	Screened G1 animals	GFP**+**	GFP**-**	GTR (%)
1	+	F	v^{1} $xdh1^{\Delta2}$	49	0	49	0
2	+	F	v^{1} $xdh1^{\Delta2}$	41	0	41	0
3	+	F	v^{1} $xdh1^{\Delta2}$	died	0	0	NA
4	+	F	v^{1} $xdh1^{\Delta2}$	died	0	0	NA
5	+	F	v^{1} $xdh1^{\Delta2}$	25	19	6	76.0
6	+	M	v^{1} $xdh1^{\Delta2}$	26	0	26	0
7	+	M	v^{1} $xdh1^{\Delta2}$	44	0	44	0
8	+	F	v^{1} $xdh1^{\Delta2}$	42	16	26	38.1
9	+	M	v^{1} $xdh1^{\Delta2}$	sterile	0	0	NA
10	+	M	v^{1} $xdh1^{\Delta2}$	5	5	0	100.0
G1 indiv#	EGFP expression	sex	crossed with	Screened G2 animals	GFP**+**	GFP**-**	G2 segregation rate (%)
5A	+	F	v^{1} $xdh1^{\Delta2}$	33	27	6	81.8
8A	+	F	v^{1} $xdh1^{\Delta2}$	64	32	32	50.0

mRNA	donor plasmid						
3xNLS-hyPBase#2	9xP3-EGFP-SV40T;attP						
G0 indiv#	EGFP expression	sex	crossed with	Screened G1 animals	GFP**+**	GFP**-**	GTR (%)
1	+	F	*v^1 $xdh1^{\Delta2}$*	48	0	48	0
2	+	M	*v^1 $xdh1^{\Delta2}$*	31	0	31	0
3	+	F	*v^1 $xdh1^{\Delta2}$*	45	31	14	68.9
4	+	M	*v^1 $xdh1^{\Delta2}$*	39	0	39	0
5	+	M	*v^1 $xdh1^{\Delta2}$*	33	6	27	18.2
G1 indiv#	EGFP expression	sex	crossed with	Screened G2 animals	GFP**+**	GF**P-**	G2 segregation rate (%)
3A	+	M	*v^1 $xdh1^{\Delta2}$*	44	37	7	84.1
3B	+	F	*v^1 $xdh1^{\Delta2}$*	51	37	14	72.5

Table S6. Results of crossing experiments for Gbact-H2B-mScarlet3 strain. F, female. GTR, germ line transformation rate.

mRNA	donor plasmid						
1xNLS-hyPBase	Gbact-H2B-mScarlet3-SV40T						
G0 indiv#	mScarlet-I expression	sex	crossed with	Screened G1 animals	RFP**+**	RF**P-**	GTR (%)
1	+	F	v^{1} $xdh1^{\Delta 2}$	30	14	16	46.7%

Table S7. Results of crossing experiments for Gbact-GAP43-mScarlet3;9xP3-EGFP strains. F, female. M, male. GTR, germ line transformation rate.

mRNA	donor plasmid						
1xNLS-hyPBase	Gbact-GAP43-mScarlet3-SV40T;9xP3-EGFP-SV40T						
G0 indiv#	EGFP expression	sex	crossed with	Screened G1 animals	GFP**+**	GFP**-**	GTR (%)
1	+	M	*v^1 $xdh1^{\Delta2}$*	45	27	18	60.0
2	+	M	*v^1 $xdh1^{\Delta2}$*	died	0	0	NA
3	+	M	*v^1 $xdh1^{\Delta2}$*	died	0	0	NA
4	+	M	*v^1 $xdh1^{\Delta2}$*	died	0	0	NA
5	+	M	*v^1 $xdh1^{\Delta2}$*	35	0	35	
6	+	M	*v^1 $xdh1^{\Delta2}$*	36	0	36	
7	+	M	*v^1 $xdh1^{\Delta2}$*	33	0	33	
8	+	F	*v^1 $xdh1^{\Delta2}$*	42	23	19	54.8
9	+	F	*v^1 $xdh1^{\Delta2}$*	35	0	35	
10	+	F	*v^1 $xdh1^{\Delta2}$*	died	0	0	NA
11	+	F	*v^1 $xdh1^{\Delta2}$*	44	19	25	43.2
G1 indiv#	EGFP expression	gender	crossing with	Screened G2 animals	GFP**+**	GFP**-**	G2 segregation rate (%)
1A	+	M	*v^1 $xdh1^{\Delta2}$*	56	28	28	50.0
1B	+	M	*v^1 $xdh1^{\Delta2}$*	46	21	25	45.7

8A	+	M	*v*[1] *xdh1*[Δ2]	45	23	22	51.1

004

Table S8. Results of crossing experiments for Ofact-H2B-mScarlet3 strain. F, female. M, male. GTR, germ line transformation rate.

mRNA	donor plasmid
3xNLS-hyPBase	Ofact-H2B-mScarlet3-SV40T

G0 indiv#	RFP expression	sex	crossed with	Screened G1 animals	RFP+	RFP-	GTR (%)
1	+	F	v^{1} $xdh1^{\Delta 2}$	20	0	20	0
2	+	F	v^{1} $xdh1^{\Delta 2}$	32	17	15	53.1
3	+	F	v^{1} $xdh1^{\Delta 2}$	20	0	20	0
4	+	F	v^{1} $xdh1^{\Delta 2}$	49	0	49	0
**G1 indiv#**	**RFP expression**	**sex**	**crossed with**	**Screened G2 animals**	**RFP+**	**RFP-**	**G2 segregation rate (%)**
1A	+	F	v^{1} $xdh1^{\Delta 2}$	50	32	18	64.0
1B	+	M	v^{1} $xdh1^{\Delta 2}$	sterile	0	0	NA
1C	+	M	v^{1} $xdh1^{\Delta 2}$	sterile	0	0	NA
1D	+	M	v^{1} $xdh1^{\Delta 2}$	31	14	17	45.2
1E	+	F	v^{1} $xdh1^{\Delta 2}$	sterile	0	0	NA
1F	+	F	v^{1} $xdh1^{\Delta 2}$	46	24	22	52.2

mRNA	donor plasmid
3xNLS-hyPBase	Ofact-H2B-mScarlet3-SV40T

G0 indiv#	RFP expression	sex	crossed with	Screened G1 animals	RFP+	RFP-	GTR (%)
1	+	F	v^1 $xdh1^{\Delta2}$	30	0	30	0.0
2	+	M	v^1 $xdh1^{\Delta2}$	51	10	41	19.6

G1 indiv#	RFP expression	sex	crossed with	Screened G2 animals	RFP+	RFP-	G2 segregation rate (%)
1A	+	F	v^1 $xdh1^{\Delta2}$	53	26	27	49.1
1B	+	M	v^1 $xdh1^{\Delta2}$	sterile	0	0	NA
1C	+	M	v^1 $xdh1^{\Delta2}$	55	33	22	60.0

mRNA	donor plasmid
3xNLS-hyPBase	Ofact-H2B-mScarlet3-SV40T

G0 indiv#	RFP expression	sex	crossed with	Screened G1 animals	RFP+	RFP-	GTR (%)
1	+	M	v^1 $xdh1^{\Delta2}$	died	0	0	NA
2	+	M	v^1 $xdh1^{\Delta2}$	died	0	0	NA
3	+	M	v^1 $xdh1^{\Delta2}$	no data	3	no data	no data

G1 indiv#	RFP expression	sex	crossed with	Screened G2 animals	RFP+	RFP-	G2 segregation rate (%)
3A, 3B, 3C	+	F (3)	v^1 $xdh1^{\Delta2}$	63	32	31	50.8

Table S9. Primers used in the study.

Name	Sequence (5'- to -3')	Purpose
Of-xdh1-F1	GGCTAACTTCCTAATGGTGGGTT	Genotyping of *Of-xdh1*
Of-xdh1-R1	CTGCCATAGCAGAAAGAAAGGT	Genotyping of *Of-xdh1*
SPLNK-BOT	CGAAGAGTAACCGTTGCTAGGAGAGACCGTGGCTGAATGAGACTGGTGTCGACACTAGTGG	Annealing of splinkerette PCR
SPLNK-GATC-TOP	GATCCCACTAGTGTCGACACCAGTCTCTAATTTTTTTTTTCAAAAAAA	Annealing of splinkerette PCR
SPLNK#1	CGAAGAGTAACCGTTGCTAGGAGAGACC	1st PCR of splinkerette PCR
SPLNK#2	GTGGCTGAATGAGACTGGTGTCGAC	2nd PCR of splinkerette PCR
5'SPLNK-PB#1	ACCGCATTGACAAGCACG	1st PCR of splinkerette PCR
5'SPLNK-PB#2	CTCCAAGCGGCGACTGAG	2nd PCR of splinkerette PCR
5'SPLNK-PB-SEQ	CGACTGAGATGTCCTAAATGC	Sequencing for splinkerette PCR

bioRxiv preprint doi: https://doi.org/10.64898/2026.07.30.741915; this version posted July 31, 2026. The copyright holder for this preprint (which was not certified by peer review) is the author/funder, who has granted bioRxiv a license to display the preprint in perpetuity. It is made available under a CC-BY 4.0 International license.

Supplementary Video Legends

Supplemental Video 1. Ventral view of a developing *O. fasciatus* embryo showing ubiquitous expression of H2B:mScarlet driven by the endogenous actin promoter identified herein. Over this 36 hour time course, katatrepsis (second inversion of the embryonic axes such that the head of the embryo moves from the posterior of the egg (right) to the anterior of the egg (left) (Panfilio, 2008)), and appendage elongation occur. The Green Fire Blue look up table implemented in Fiji (Schindelin et al., 2012) shows the highest intensity pixels in white, intermediate intensity pixels in green, and the lowest intensity pixels in black (see Figure 5D). Download Link

Supplemental Video 2. Dorsal view of a developing *O. fasciatus* embryo showing ubiquitous expression of H2B:mScarlet driven by the endogenous actin promoter identified herein. Over this 36 hour time course, the end of anatrepsis (first inversion of the embryonic anteroposterior axis such that the head of the embryo moves from the anterior of the egg (right) to the posterior of the egg (left)), and the whole of katatrepsis (second inversion of both anteroposterior and dorsoventral embryonic axes relative to those of the egg) occur (Panfilio, 2008). The Green Fire Blue look up table implemented in Fiji (Schindelin et al., 2012) shows the highest intensity pixels in white, intermediate intensity pixels in green, and the lowest intensity pixels in black (see Figure 5D). Download Link

Supplemental Video 3. Lateral view of a developing *O. fasciatus* embryo showing ubiquitous expression of H2B:mScarlet driven by the endogenous actin promoter identified herein. Over this 36 hour time course, the end of anatrepsis and the whole of

bioRxiv preprint doi: https://doi.org/10.64898/2026.07.30.741915; this version posted July 31, 2026. The copyright holder for this preprint (which was not certified by peer review) is the author/funder, who has granted bioRxiv a license to display the preprint in perpetuity. It is made available under a CC-BY 4.0 International license.

katatrepsis (Panfilio, 2008) occur as in Supplemental Video 3. From this lateral viewpoint, emerging muscular contractions of the embryo are apparent beginning at approximately 30 hours, the elongating appendages on the ventral side of the embryo (bottom), and the dorsal movement (towards the top) of the lateral ectoderm that is required for dorsal closure, are evident. The Green Fire Blue look up table implemented in Fiji (Schindelin et al., 2012) shows the highest intensity pixels in white, intermediate intensity pixels in green, and the lowest intensity pixels in black (see Figure 5D).

Download Link
